# B microchromosomes in the family Curimatidae (Characiformes): mitotic and meiotic behavior

**DOI:** 10.3897/CompCytogen.v5i4.1650

**Published:** 2011-11-09

**Authors:** Tatiane Ramos Sampaio, Waleska Gravena, Juceli Gonzalez Gouveia, Lucia Giuliano-Caetano, Ana Lúcia Dias

**Affiliations:** 1 Laboratório de Citogenética Animal, Centro de Ciências Biológicas, Departamento de Biologia Geral, CCB, Universidade Estadual de Londrina, Rodovia Celso Garcia Cid, PR 445, Km 380, CEP 86051-970, P.O. Box 6001, Londrina, Paraná, Brasil.; 2 Laboratório de Evolução e Genética Animal, Instituto de Ciências Biológicas, Universidade Federal do Amazonas, Av. General Rodrigo Otávio, n.3000, Japiim II CEP 69070-000 Manaus, Amazonas, Brasil

**Keywords:** B microchromosome, meiosis, curimatids

## Abstract

In the present work, six curimatid species were analyzed: *Cyphocharax voga* (Hensel, 1870), *Cyphocharax spilotus* (Vari, 1987), *Cyphocharax saladensis* (Meinken, 1933), *Cyphocharax modestus* (Fernández-Yépez, 1948), *Steindachnerina biornata* (Braga & Azpelicueta, 1987) and *Steindachnerina insculpta* (Fernández-Yépez, 1948) collected from two hydrographic basins. All samples presented 2n=54 meta-submetacentric (m-sm) chromosomes and FN equal to 108, and 1 or 2 B microchromosomes in the mitotic and meiotic cells of the six sampled populations showing inter-and intraindividual variation. The analysis of the meiotic cells in *Cyphocharax saladensis*, *Cyphocharax spilotus*, and *Cyphocharax voga* showed a modal number of 54 chromosomes in the spermatogonial metaphases and 27 bivalents in the pachytene, diplotene, diakinesis and in metaphase I stages, and 27 chromosomes in metaphase II; in *Cyphocharax modestus*, *Steindachnerina biornata*, and *Steindachnerina insculpta*, spermatogonial metaphases with 54 chromosomes and pachytene and metaphase I with 27 bivalents were observed. The B microchromosome was observed as univalent in the spermatogonial metaphase of *Cyphocharax spilotus*, in the pachytene stage in the other species, with the exception of *Cyphocharax saladensis*, and *Steindachnerina biornata* in metaphase I. New occurrences of the B microchromosome in *Cyphocharax voga*, *Cyphocharax saladensis* and *Steindachnerina biornata* were observed, confirming that the presence of this type of chromosome is a striking characteristic of this group of fish.

## Introduction

B chromosomes, also known as supernumerary or accessory chromosomes, are additional dispensable chromosomes present in some individuals of some populations in some species. They have probably originated from the A complement, but followed their own evolutionary paths, being found in different groups of both animals and plants (Camacho et al. 2000).

The irregular behavior of this chromosome type in mitosis and in meiosis causes it to accumulate selfishly in the germ line of many species, producing a non-Mendelian segregation with transmission rates higher than those yielded by the chromosomes of the A complement (Camacho et al. 2000). B chromosomes present in an individual can exhibit a parasitic, neutral or beneficial behavior (Jones and Rees 1982).

In freshwater Neotropical fish, the occurrence of B chromosomes has been reported in 61 species, distributed in 16 families of seven different orders and in distinct hydrographic basins, according with the revision accomplished by Carvalho et al. (2008). The order Characiformes possesses the majority of the species bearing B chromosomes, including 31 species of six different families: Anostomidae, Characidae, Crenuchidae, Curimatidae, Parodontidae and Prochilodontidae.

The first work to record the presence of the B chromosome in the family Curimatidae was carried out by Venere and Galetti (1985) in an individual of *Cyphocharax modestus* (Fernández-Yépez, 1948) collected from the Tiete River, municipality of Águas de São Pedro/SP, which proved to be entirely heterochromatic. Since then, other populations of *Cyphocharax modestus* and other species, such as *Cyphocharax spilotus* (Vari, 1987), *Cyphocharax gouldingi* Vari, 1992 and *Steindachnerina insculpta* (Fernández-Yépez, 1948) have shown the presence of this extra chromosome (Gravena et al. 2007; Venere et al. 2008).

The current study examines the frequency, behavior and distribution of B microchromosomes in mitotic and meiotic cells in six fish species of the family Curimatidae from two hydrographic basins.

## Material and methods

**Figure 1.**
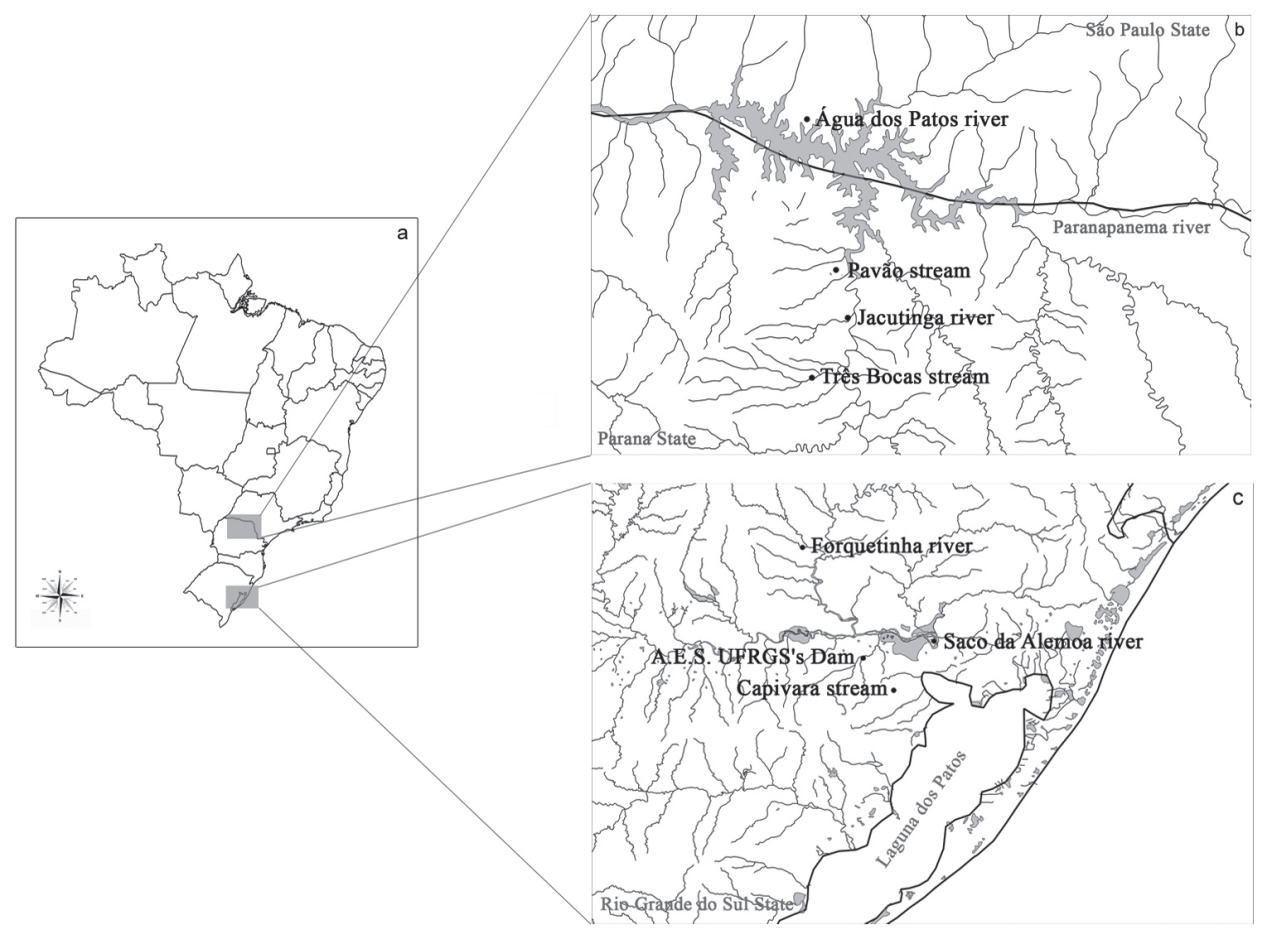
**a** Map of Brazil **b** Collection sites of Paranapanema River basin: Água dos Patos River in the São Paulo state, Pavão stream, Jacutinga River and Tres Bocas stream in the Parana state **c** Collection sites of Laguna dos Patos Hydrographic System: Forquetinha River, Saco da Alemoa River, Agronomic Experiment Station of UFRGS’s Dam and Capivara stream in the Rio Grande do Sul state.

Six species of the family Curimatidae were analysed: *Cyphocharax voga* (Hensel, 1870), *Cyphocharax spilotus* (Vari, 1987), *Cyphocharax saladensis* (Meinken, 1933), *Cyphocharax modestus* (Fernández-Yépez, 1948), *Steindachnerina biornata* (Braga & Azpelicueta, 1987)and *Steindachnerina insculpta* (Fernández-Yépez, 1948), collected from the Laguna dos Patos Hydrographic System/RS and Paranapanema River basin/SP/PR (Fig. 1, Table 1). Voucher specimens are catalogued in the Zoology Museum of the Universidade Estadual de Londrina, Paraná state, under catalog numbers: MZUEL 5105 – *Cyphocharax voga*; MZUEL 5106 – *Cyphocharax spilotus*; MZUEL 5058 – *Cyphocharax saladensis*; MZUEL 1374 – *Cyphocharax modestus*; MZUEL 5059 – *Steindachnerina biornata* and MZUEL 1042 – *Steindachnerina insculpta*.

**Table 1.** Species analysed, collection sites and hydrographic basins.

**Species**	**Number of individuals**	**Collection sites**	**Basins**
*Cyphocharax voga*	1♀, 1♂	Saco da Alemoa River, Eldorado do Sul, RS, Brazil / S29°59'15.6", W51°14'24.1"	Laguna dos Patos Hydrographic System
	2♀, 9♂	Capivara stream, Barra do Ribeiro, RS, Brazil / S30°17'33.3", W51°19'23.6"	
*Cyphocharax spilotus*	2♀, 3♂	Capivara stream, Barra do Ribeiro, RS, Brazil / S30°17'33.3", W51°19'23.6"	
*Cyphocharax saladensis*	1♀, 10♂	Agronomic Experiment Station of UFRGS’s Dam, Eldorado do Sul, RS, Brazil / S30°05'36.2", W51°40'41.8"	
*Steindachnerina biornata*	1♀, 1♂	Forquetinha River, Canudos do Vale, RS, Brazil / S29°19'20.9", W50°14'3.6"	
*Cyphocharax modestus*	2♀, 5♂	Tres Bocas stream, Londrina, PR, Brazil / S23°17'12.9", W51°13'58.2"	Paranapanema River
*Steindachnerina insculpta*	3♂	Tres Bocas stream, Londrina, PR, Brazil / S23°17'12.9", W51°13'58.2"	
	2♂	Pavão stream, Sertanópolis, PR, Brazil	
		4♀, 8♂		Jacutinga River, Londrina, PR, Brazil / S23°23'6.6", W51°04'35.8"
				1♀, 5♂	Água dos Patos River, Iepê, SP, Brazil / S22°41'17.7", W51°05'23.9"

Mitotic chromosomes were obtained by direct preparation removing the anterior kidney, according to Bertollo et al. (1978) and meiotic chromosomes were obtained using gonadal cells by technique developed by Kligerman and Bloom (1977), with modifications. Chromosomes were characterized as metacentric (m) and submetacentric (sm), according to Levan et al. (1964).

## Results and discussion

**Figure 2.**
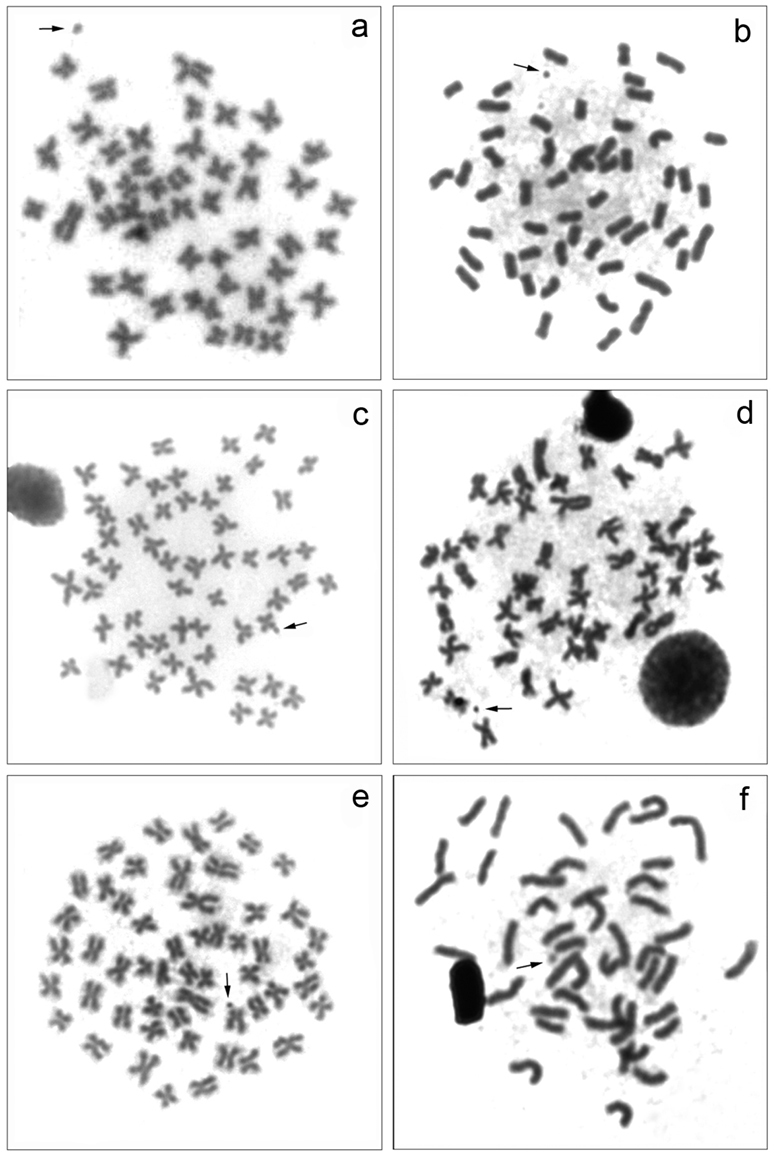
Somatic metaphases: **a** *Cyphocharax voga* **b** *Cyphocharax spilotus* **c** *Cyphocharax saladensis* **d** *Cyphocharax modestus* **e** *Steindachnerina biornata* **f** *Steindachnerina insculpta*. The arrows indicate the B microchromosome.

All samples analyzed showed a diploid number of 54 meta-submetacentric chromosomes (m-sm) and a fundamental number (FN) equal to 108 (Fig. 2). This karyotype structure is often found in this fish group, and are conservative among the species of the family Curimatidae, as already observed by Brassesco et al. (2004) and Venere et al. (2008). Among the populations studied, *Cyphocharax voga* and *Cyphocharax spilotus* collected in Capivara stream/RS and *Cyphocharax modestus* and *Steindachnerina insculpta* collected in Três Bocas stream/PR are living in sympatry.

One B microchromosome was observed in all populations studied, with variation in the number and frequency among them (Fig. 2). In the species *Cyphocharax voga*, *Cyphocharax spilotus*, *Cyphocharax saladensis* and *Steindachnerina biornata* belonging to the Laguna dos Patos Hydrographic System, there was an inter-and intraindividual variation from 0 to 1 B microchromosome in the somatic cells (Table 2). In *Cyphocharax modestus* and *Steindachnerina insculpta*, from the Paranapanema River basin, up to two B microchromosomes, also exhibiting inter-and intraindividual variation, were detected in the somatic cells (Table 3). As proposed by Jones and Rees (1982), these variations among species represent a mitotic instability of this chromosome, probably due to its non-Mendelian behavior during cell division.

**Table 2.** B microchromosome frequency in somatic cells of the curimatids from Laguna dos Patos Hydrographic System/RS.

**Species**	**Locality**	**Specimens**	**Sex**	**Number of B chromosome**		**Total number of cells**
				**0**	**1**	
*Cyphocharax voga*	Saco da Alemoa River	149	♀	22	2	24
		150	♂	3	0	3
		**Total / %**		**25 / 92,6**	**2 / 7,4**	**27**
	Capivara stream	748	♀	3	1	4
		752	♂	4	1	5w
		755	♀	17	0	17
		777	♂	42	1	43
		780	♂	12	0	12
		**Total / %**		**78 / 96,3**	**3 / 3,7**	**81**
*Cyphocharax spilotus*	Capivara stream	580	♂	2	0	2
		753	♀	25	3	28
		758	♀	23	0	23
		778	♂	8	1	9
		779	♂	22	0	22
		**Total / %**		**80 / 95,2**	**4 / 4,8**	**84**
*Cyphocharax saladensis*	Agronomic Experiment Station of UFRGS’s Dam	784	♂	4	0	4
		786	♀	5	0	5
		787	♂	6	0	6
		788	♂	36	1	37
		789	♂	10	0	10
		790	♂	8	0	8
		791	♂	7	2	9
		792	♂	3	0	3
		793	♂	2	0	2
		794	♂	6	0	6
		**Total / %**		**87 / 96,7**	**3 / 3,3**	**90**
*Steindachnerina biornata*	Forquetinha River	857	♀	55	3	58
		996	♂	3	2	5
		**Total / %**		**58 / 92**	**5 / 8**	**63**

**Table 3.** B microchromosome frequency in somatic cells of the curimatids from Paranapanema River basin.

**Species**	**Locality**	**Specimens**	**Sex**	**Number of B chromosome**			**Total number of cells**
				**0**	**1**	**2**	
*Cyphocharax modestus*	Tres Bocas stream	2656	♂	5	0	0	5
		3815	♂	18	0	0	18
		3909	♀	8	0	0	8
		3992	♀	46	3	1	50
		**Total / %**		**77 / 95**	**3 / 3,75**	**1 / 1,25**	**81**
*Steindachnerina insculpta*	Pavão stream	3277	♂	3	2	0	5
		3278	♂	8	0	0	8
		**Total / %**		**11 / 84,6**	**2 / 15,4**	**0 / 0**	**13**
	Água dos Patos River	3393	♀	40	8	0	48
		3407	♂	18	0	0	18
		3408	♂	11	2	1	14
		3409	♂	21	0	0	21
		3411	♂	22	0	0	22
		3745	♂	5	0	0	5
		**Total / %**		**117 / 91,4**	**10 / 7,8**	**1 / 0,8**	**128**
	Jacutinga River	3453	♀	15	2	1	18
		3454	♀	22	0	0	22
		3461	♂	14	0	0	14
		3462	♂	20	0	0	20
		3465	♂	23	1	0	24
		3862	♀	6	0	0	6
		3986	♂	2	0	0	2
		3987	♂	5	0	0	5
		3991	♂	4	0	0	4
		3993	♀	3	0	0	3
		4046	♂	8	0	0	8
		4049	♂	4	10	0	14
		**Total / %**		**126 / 90**	**13 / 9,3**	**1 / 0,7**	**140**

Of the total number of somatic cells with B microchromosomes analyzed in six species of Curimatids, there was a variation from 3.3% in *Cyphocharax saladensis* to 15.4% in *Steindachnerina insculpta*. Among the species belonging to the Laguna dos Patos Hydrographic System, *Cyphocharax voga* showed the highest percentage of B cells (11.1%), followed by *Steindachnerina biornata* with 8%, *Cyphocharax spilotus* with 4.8%, and *Cyphocharax saladensis* with 3.3% (Table 2).

In the Paranapanema River basin, the population of the *Steindachnerina insculpta* from the Pavão stream/PR showed 15.4% of their somatic cells with B microchromosomes, followed by the populations of the Jacutinga River/PR with 10% and Água dos Patos River/SP with 8.6%. The species *Cyphocharax modestus* from the Tres Bocas stream/PR presented 5% of their cells with B microchromosomes (Table 3). The data collected from both basins corroborate the constant presence of this type of chromosome in the Curimatidae family, constituting a striking characteristic of the group, even when its incidence is low.

Specimens of *Cyphocharax voga* collected at two localities in the Laguna dos Patos Hydrographic System (Saco da Alemoa River and Capivara stream) not presented interpopulation differences in the number and frequency of the Bs. Likewise were not observed significant differences between the four populations of *Steindachnerina insculpta*, belonging to Paranapanema River basin.

The B microchromosome was observed in four species of curimatids collected from different populations: *Cyphocharax gouldingi* (Venere et al. 2008), *Cyphocharax modestus* (Gravena et al. 2007), *Cyphocharax spilotus* (Brassesco et al. 2004), *Steindachnerina insculpta* (Gravena et al. 2007), and three new species assessed in this study: *Cyphocharax saladensis*, *Cyphocharax voga* and *Steindachnerina biornata*, representing 18.42% of all species studied, always small in size with inter and intra individual variation (Table 4). Among these, *Cyphocharax modestus* and *Steindachnerina insculpta* are the species that possess B microchromosomes in all populations studied, besides being the species that have the widest range of cytogenetic studies to date.

**Table 4.** Cytogenetic date of differents species of curimatids (2n: diploid number; FN: number fundamental; Bs: supernumerary chromosomes).

**Species**	**2n**	**FN**	**Bs**	**B Size**	References*
*Curimata cyprinoides*	54	108	-	-	3, 15
*Curimata inornata*	54	108	-	-	3, 15
*Curimata kneri*	54	108	-	-	3
*Curimata ocellata*	56	112	-	-	3
*Curimata vittata*	54	108	-	-	3
*Curimatella alburna*	54	108	-	-	3
*Curimatella dorsalis*	54	108	-	-	8, 12
*Curimatella imaculata*	54	108	-	-	15
*Curimatella lepidura*	54	108	-	-	2
*Curimatella meyeri*	54	108	-	-	3
*Curimatopsis myersi*	46	46	-	-	8
*Cyphocharax gilbert*	54	108	-	-	6, 15
*Cyphocharax* cf. *gillii*	54	108	-	-	2
*Cyphocharax gouldingi*	54	108	0 - 1	Micro	15
*Cyphocharax modestus*	54	108	0 - 4	Micro	1, 2, 7, 9, 13, 14, 16, 17,18
*Cyphocharax nagelii*	54	108	-	-	2, 15
*Cyphocharax* cf. *spilurus*	54	108	-	-	2
*Cyphocharax spilotus*	54	108	0 - 1	Micro	11, 12, 18
*Cyphocharax vanderi*	54	108	-	-	2
*Cyphocharax voga*	54	108	0 - 1	Micro	2, 12, 18
*Cyphocharax platanus*	58	116	-	-	12, 15
*Cyphocharax saladensis*	54	108	0 - 1	Micro	18
*Potamorhina altamazonica*	102	106	-	-	4
*Potamorhina latior*	56	112	-	-	4
*Potamorhina pristigaster*	54	108	-	-	4
*Potamorhina squamoralevis*	102	106	-	-	12
*Psectrogaster amazonica*	54	108	-	-	15
*Psectrogaster curviventris*	54	108	-	-	8, 12
*Psectrogaster rutiloides*	54	108	-	-	3
*Steindachnerina amazonica*	54	108	-	-	15
*Steindachnerina biornata*	54	108	0 - 1	Micro	18
*Steindachnerina brevipina*	54	108	-	-	8, 12
*Steindachnerina conspersa*	54	108	-	-	2, 12
*Steindachnerina elegans*	54	108	-	-	2
*Steindachnerina gracilis*	54	108	-	-	15
*Steindachnerina* cf. *guentheri*	54	108	-	-	10
*Steindachnerina insculpta*	54	108	0 - 2	Micro	2, 5, 13, 14, 15, 17,18
*Steindachnerina leucisca*	54	108	-	-	3

**References:** **1** Venere and Galetti (1985) **2** Venere, Galetti (1989) **3** Feldberg et al. (1992) **4** Feldberg et al. (1993) **5** Oliveira and Foresti (1993) **6** Venerea and Galetti Jr. (1995) **7** Martins et al. (1996) **8** Navarrete and Júlio-Jr. (1997) **9** Venere et al. (1999) **10** Carvalho et al. (2001) **11** Fenocchio et al. (2003) **12** Brassesco et al. (2004) **13** Gravena et al. (2007) **14** Teribele et al. (2008) **15** Venere et al. (2008) **16** De Rosa et al. (2007) **17** De Rosa-Santos et al. (2008) **18** present study.

Camacho et al. (2000), reported that differences in the incidence of B chromosomes among populations depend on selection factors (such as relationship between the Bs and the environmental conditions, including temperature and altitude), historical factors (such as number of generations since the origin of Bs in the population or even in the species), transmission factors (in relation to the mechanisms of accumulation), and random factors. These four types of factors, which are likely to act simultaneously, make it difficult to evaluate the action of each one separately, even when a more detailed study of each species occurs.

**Figure 3.**
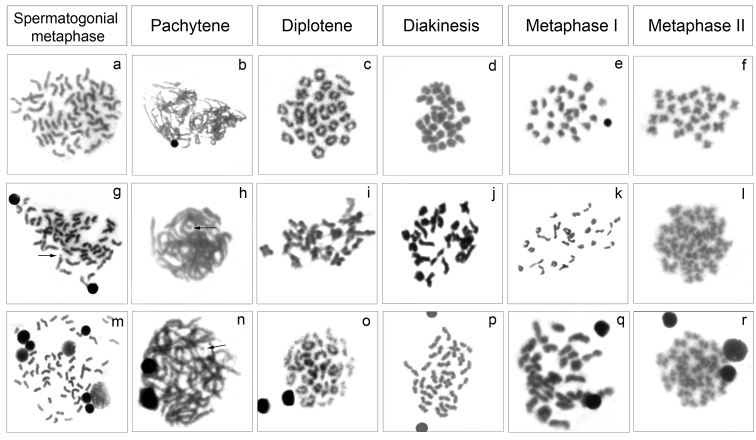
Meiotic cells: *Cyphocharax saladensis* **a–f** *Cyphocharax spilotus* **g–l** and *Cyphocharax voga* **m–r **belonging to Laguna dos Patos Hydrographic System. The arrows indicate the B microchromosome univalent in **g,** **h** and **n**.

**Figure 4.**
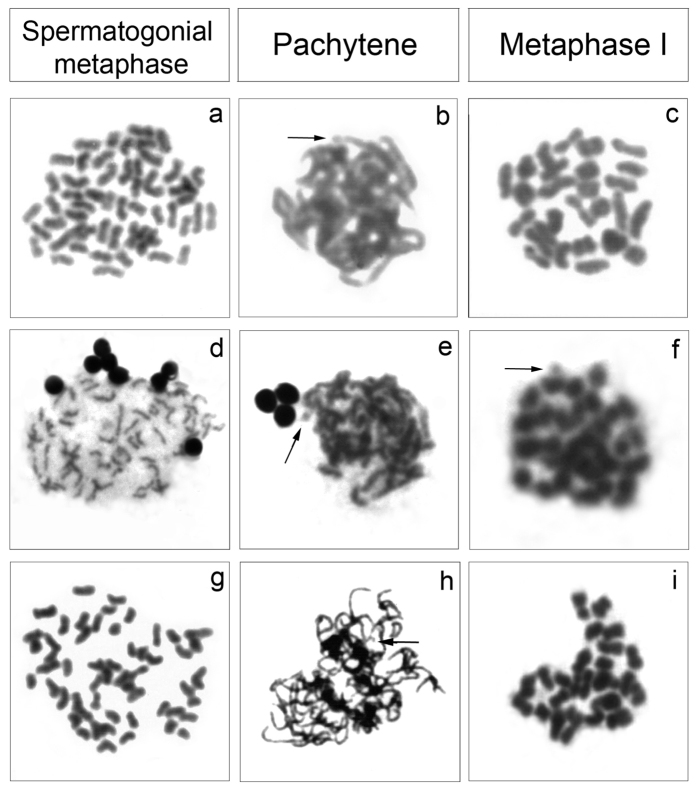
Meiotic cells: *Cyphocharax modestus* **a–c**, *Steindachnerina biornata* **d–f** and *Steindachnerina insculpta* **g–i**. The arrows indicate the B microchromosome univalent in **b,** **e,** **f** and **h**.

The analysis of meiotic cells in *Cyphocharax saladensis*, *Cyphocharax spilotus* and *Cyphocharax voga* showed a modal number of 54 chromosomes in spermatogonial metaphases and 27 bivalents in the stages of pachytene, diplotene, diakinesis and metaphase I, and 27 chromosomes in metaphase II (Fig. 3). In *Cyphocharax modestus*, *Steindachnerina biornata* and *Steindachnerina insculpta*, spermatogonial metaphases with 54 chromosomes and pachytene and metaphase I with 27 bivalents were observed (Fig. 4). It was possible to observe the B microchromosome as univalent in the spermatogonial metaphase in *Cyphocharax spilotus*; in the pachytene stage in *Cyphocharax spilotus*, *Cyphocharax voga*, *Cyphocharax modestus*, *Steindachnerina biornata* and *Steindachnerina insculpta*; and in metaphase I in *Steindachnerina biornata* (Figs 3, 4).

In both types of cell division, the number of cells without B microchromosomes was greater than number of cells with B microchromosomes in the species of Curimatidae. Camacho et al. (2000) suggest that the small number of chromosomes in diploid cells represents the maximum that a species is able to tolerate as adults.

In others groups of fishes with B-chromosomes meiotic analysis has been performed in order to understand the behavior of this chromosome, as in *Prochilodus lineatus* (Valenciennes, 1836) from the Mogi Guaçu River (Pirassununga/SP), whose studies of the synaptonemal complex showed that no B chromosome paired with autosomal chromosomes. In the late pachytene stage, 27 paired bivalents and small bivalent, trivalent and quadrivalent B chromosomes were observed. The pairing of B chromosomes was interpreted as a result of homology between these chromosomes (Dias et al. 1998).

Borin and Martins-Santos (2004) analyzed *Pimelodus* sp. and *Pimelodus ortmanni* Haseman, 1911 from the Iguaçu River, in the Parana state, which had 2n=56 and intraindividual variations from 0 to 4 B chromosomes in the somatic cells. The meiotic analysis confirmed the presence of these chromosomes, with a variation ranging from 0 to 2 B chromosomes in metaphase I, but could not confirm whether these Bs were univalent or bivalent. The species *Rineloricaria pentamaculata* Langeani & Araujo, 1994 from the Tauá stream, Parana River basin, studied by Porto et al. (2010), also showed a variation in the diploid number from 56 to 59 chromosomes, attributed to the presence of B chromosomes, which ranged from 0 to 3 in the somatic cells, and confirmed by the meiotic analysis that showed 28 bivalents in metaphases I and II and small univalents. These data support the classification of such elements as supernumerary or B chromosomes, indicating meiotic instability in the transmission to the offspring (Porto et al. 2010).

The meiotic data presented in this study are the first for Curimatidae, and also indicate the instability of the B microchromosome during meiosis, demonstrating that this chromosome has no homology with any normal chromosome complement in these species. Analyses of the synaptonemal complex in the analyzed species would be interesting to complement the study of the meiotic behavior of B microchromosome in the species Curimatidae.

According Camacho et al. (2000), these chromosomes could be originated from the A chromosomes (intraspecific origin) or as result of mating between species (interspecific origin). Some authors discuss the origin of the B chromosomes in different species of fish as in *Astyanax scabripinnis* (Jenyns 1842) (Moreira-Filho et al. 2004), Amazon species cichlids (Feldberg et al. 2004) and *Rhamdia quelen* (Quoy & Gaimard, 1824) (Moraes et al. 2009).

There are two hypotheses that could explain the origin of B chromosomes in *Cyphocharax modestus* and *Steindachnerina insculpta*, according Martins et al. (1996). The first one suggests that these chromosomes arose in some ancestor of the family and were eliminated in species where they are not found today. The second one suggests that B chromosomes have a more recent and independent origin in the species that bear it.

The results obtained in this study provides more information about the occurrence of B microchromosomes in the curimatids, confirming its presence in *Cyphocharax spilotus*, *Cyphocharax modestus* and *Steindachnerina insculpta*, previously described in other populations, and showing new events in *Cyphocharax voga*, *Cyphocharax saladensis* and *Steindachnerina biornata*. These dataconfirm the outstanding characteristic of this type of chromosome in this group of fish and its mitotic and meiotic instability and allow a further discussion about the origin of Bs in the family Curimatidae.
